# Real world time trends in antithrombotic treatment for newly diagnosed atrial fibrillation in China: reports from the GLORIA-AF Phase III registry: Trends in antithrombotic therapy use in China

**DOI:** 10.1186/s12959-023-00527-x

**Published:** 2023-08-01

**Authors:** Xiaoxia Liu, Guoze Feng, Sabrina Vogel Marler, Menno V Huisman, Gregory Y. H. Lip, Changsheng Ma

**Affiliations:** 1 Department of Cardiology, Beijing An Zhen Hospital, Capital Medical University, Chaoyang District, Beijing, 100029 China; 2 Boehringer Ingelheim, Shanghai, China; 3 Boehringer Ingelheim Pharmaceuticals, Inc, Ridgefield, CT USA; 4 Department of Thrombosis and Hemostasis, Leiden University Medical Center, Leiden, the Netherlands; 5 Liverpool Centre for Cardiovascular Science at University of Liverpool, Liverpool John Moores University and Liverpool Heart & Chest Hospital, Liverpool, United Kingdom; 6 Danish Center for Health Services Research, Department of Clinical Medicine, Aalborg University, Aalborg, Denmark

**Keywords:** Atrial fibrillation, Oral anticoagulation, Stroke, Prevention, China

## Abstract

**Background:**

Stroke prevention with oral anticoagulant (OAC) therapy, including non-vitamin K antagonist oral anticoagulants (NOACs), is recommended in patients with atrial fibrillation (AF). This analysis describes the antithrombotic prescription patterns for Chinese patients enrolled post-dabigatran approval during Phase II and III of the Global Registry on Long-Term Oral Antithrombotic Treatment in Patients with Atrial Fibrillation (GLORIA-AF) program in China.

**Methods:**

Patients aged ≥ 18 years with newly diagnosed (< 3 months before baseline visit) nonvalvular AF at risk of stroke (CHA_2_DS_2_-VASc score ≥ 1) were consecutively enrolled in the GLORIA-AF registry. This cross-sectional analysis provides descriptive comparison of Chinese patients in Phase III (2015–2016) with those enrolled in Phase II (2013–2014).

**Results:**

Overall, 1,018 and 1,911 Chinese patients were eligible for analysis in Phase II and III, respectively. Most patients (69.6% and 69.1%, respectively) had high stroke risk (CHA_2_DS_2_-VASc score ≥ 2 for males and ≥ 3 for females). High bleeding risk (HAS-BLED score ≥ 3) rates were similar (17.3% for Phase II, 17.6% for Phase III). In Phase II, 5.8%, 15.2%, 36.7% and 42.2% of patients were prescribed NOACs, vitamin K antagonists (VKAs), antiplatelet therapies or no antithrombotic treatment, respectively. The corresponding figures were 17.2%, 23.5%, 37.4% and 21.8% for patients in Phase III, with an overall increase in OAC prescriptions (NOACs or VKAs). In patients with high stroke risk, the prescription patterns in Phase II were 5.6%, 14.4%, 41.0% and 38.9% for NOACs, VKAs, antiplatelets or no antithrombotic treatment, respectively. The respective proportions in Phase III were 15.1%, 23.5%, 40.9% and 20.5%.

**Conclusions:**

Since the availability of dabigatran in China, the overall trend of OAC, including NOAC, prescriptions in Chinese patients with nonvalvular AF has increased over time, albeit with VKAs as the most common antithrombotic treatment. Most patients, including those at high stroke risk, remain undertreated according to best practice guidelines.

**Trial registration:**

ClinicalTrials.gov NCT01468701.

**Supplementary Information:**

The online version contains supplementary material available at 10.1186/s12959-023-00527-x.

## Background

Atrial fibrillation (AF) is increasing in prevalence and incidence, conferring a substantial risk of mortality and morbidity from ischemic stroke [1, 2], with a subsequent healthcare cost burden [3, 4]. The risk of ischemic stroke [5, 6] and intracranial hemorrhage [7] is even higher in Asian than non-Asian patients with AF. In China, the most recent prevalence rate of AF according to a nationwide community-based survey was estimated to be 1.8%, equating to nearly 8 million Chinese adults aged > 45 years with a diagnosis of AF [8]; this burden is only expected to increase with the rapidly aging population. Therefore, stroke prevention with effective therapies is an essential priority for public health.

Previously, antithrombotic therapy has been shown to reduce stroke risk by almost two-thirds in patients who have AF [9]. Local and international guidelines have since been updated to recommend oral anticoagulant (OAC) therapy, namely vitamin K antagonists (VKAs) or non-vitamin K antagonist oral anticoagulants (NOACs), as treatment for patients with AF and stroke risk factors (CHA_2_DS_2_-VASc score for AF and stroke risk ≥ 1 in males or ≥ 2 in females); aspirins have been found ineffective [6, 10–13]. The global shift from antiplatelets to OACs among patients with AF has been documented through real-world clinical databases, positively reflecting the change at a clinical level for ensuring that patients receive evidence-based treatments [14–16]. However, findings from another nationwide study in China during a similar timeframe suggest a poor uptake of anticoagulant treatment locally, with the majority of patients either receiving no treatment or being prescribed antiplatelet therapies [17].

The Global Registry on Long-Term Oral Antithrombotic Treatment in Patients with Atrial Fibrillation (GLORIA-AF; NCT01428765 [Phase I], NCT01468701 and NCT01671007 [Phases II/III]) is one of the first, large, international, prospective, observational registries to collect data on patients with newly diagnosed nonvalvular AF, enrolling patients from 38 countries across different geographic regions between 2011 and 2016, including China. The registry was designed to monitor antithrombotic treatment patterns in three phases during this time period in participating regions, collecting data from patients who enrolled before and after NOACs were available [18, 19].

This analysis aims to provide a descriptive comparison of the baseline characteristics and antithrombotic prescribing patterns of stroke prevention in patients from China diagnosed with AF, who were enrolled in the GLORIA-AF Phase II and Phase III program after dabigatran became available (approved by the China Food and Drug Administration in February 2013) and had been established as routine clinical care.

## Methods

### Design and setting

**Fig. 1:**
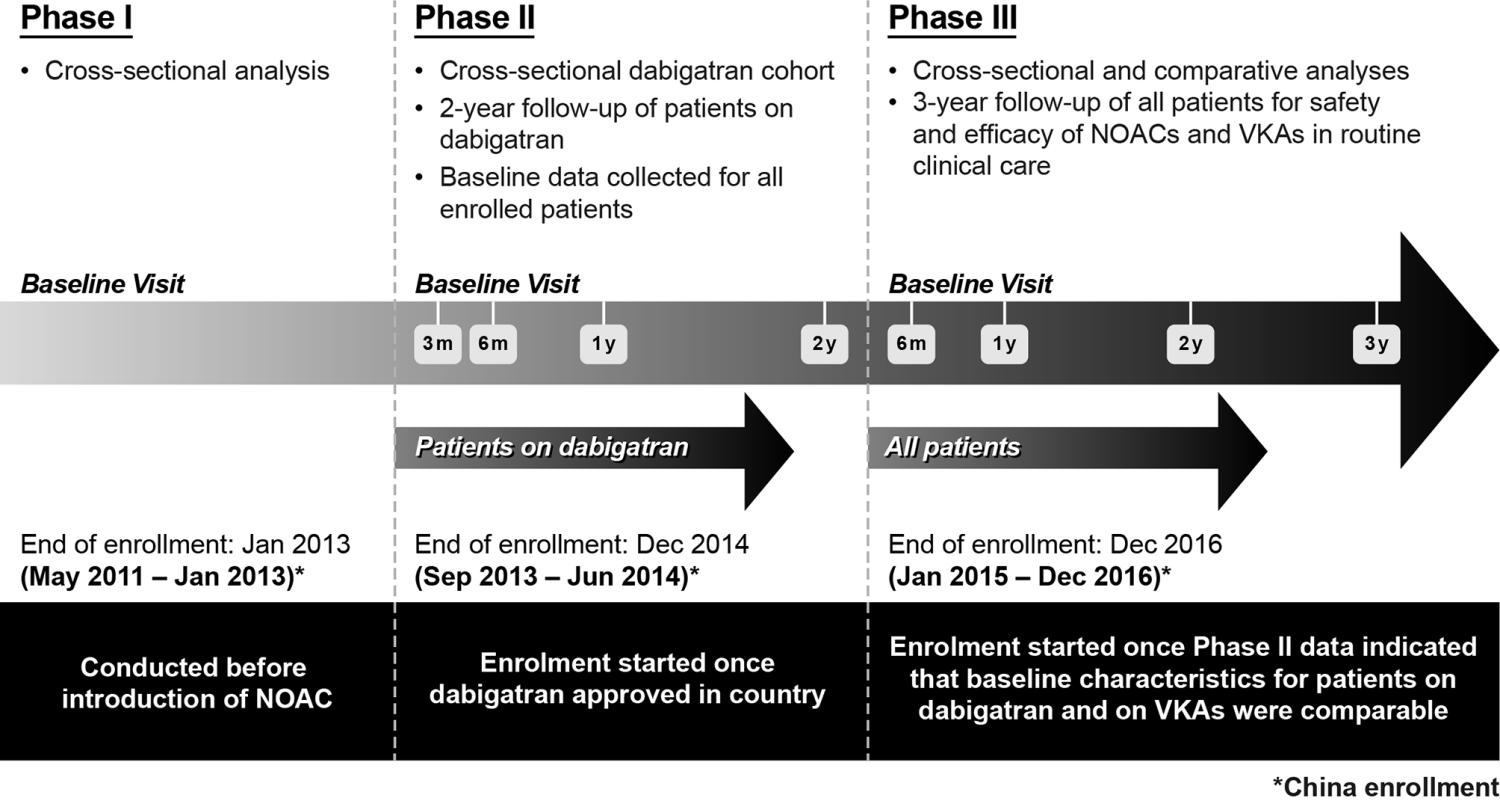
Schematic of the GLORIA-AF program GLORIA-AF, Global Registry on Long-Term Oral Antithrombotic Treatment in Patients with Atrial Fibrillation; NOAC, non-VKA oral anticoagulant; VKA, vitamin K antagonist

The full methodology for GLORIA-AF has been described previously [18]. The program is a global disease registry of newly diagnosed patients with AF, with a three-phase study design (Fig. 1) [18]. Phase I (enrollments 2011–2013) had a cross-sectional design and commenced prior to approval of dabigatran, with no data collected beyond the initial visit [18]. Phase II (enrollments 2011–2014) was initiated following approval of dabigatran in each participating country; patients who received dabigatran were followed up for 2 years, allowing case–control analyses of risk factors for different outcomes [18]. Phase III (enrollments 2014–2016) was initiated when the baseline characteristics of patients receiving dabigatran and VKAs in Phase II were sufficiently similar to allow for comprehensive comparative analysis, as determined by propensity score methodology. All patients in GLORIA-AF were managed according to local clinical practice, with treatment decisions solely at the discretion of the treating physician. Baseline characteristics data were collected at each study phase, irrespective of antithrombotic treatment status. Patient de-identified clinical data and site characteristics were captured using a standardized data collection form over a web-based system. An independent steering committee oversaw the design, execution, study conduct, and publication development.

### Patients

GLORIA-AF consecutively enrolled patients aged ≥ 18 years with newly diagnosed (< 3 months before the baseline visit) nonvalvular AF at risk for stroke (defined as CHA_2_DS_2_-VASc score ≥ 1). Exclusions included placement of any mechanical heart valve, or valve disease expected to require valve replacement intervention; patients who had received more than 60 days of VKA treatment in their lifetime; AF with a generally reversible cause; and medical conditions other than AF that required chronic use of an OAC. Patients were classified into four groups according to their prescribed antithrombotic treatment: NOACs, VKAs, antiplatelet therapies without OACs (alone), and no antithrombotic treatment. Additionally, these treatment groups were separated into two broader subgroups of OACs (NOAC or VKA), or no OACs (antiplatelets or no antithrombotic treatment). Patients were also assessed for stroke and bleeding risks using the CHA_2_DS_2_-VASc score and HAS-BLED score for major bleeding risk [20, 21].

The study was approved by the respective institutional review board or independent ethics committees of participating institutions according to national and international regulations, and was conducted in accordance with the Declaration of Helsinki International Committee on Harmonization Guidelines for Good Clinical Practice. All patients provided written informed consent.

### Statistical analyses

This was a descriptive comparative analysis of all eligible patients from China enrolled in GLORIA-AF Phase II/III, stratified by treatment group. The main outcome of interest was the choice of antithrombotic prescription. Patients receiving combination therapy of OACs were not included in this analysis. Baseline characteristics such as demographic and clinical data, medical history, AF characterizations, and risk scores for stroke and bleeding were reported. Data were summarized by mean (standard deviation) for continuous variables, and by frequencies and percentage for categorical variables. Data were also stratified by treatment group (NOAC, VKA, antiplatelet, no antithrombotic treatment), and by subgroup for bleeding and stroke risks. No inferential statistical analyses or statistical hypothesis tests were performed. Standardized differences were used to assess baseline comparability between Phase III and Phase II. All analyses were performed using SAS® software version 9.4 (SAS Institute, Inc., Cary, NC, USA).

## Results

### Baseline characteristics

Between November 2011 and February 2014, 10,871 eligible patients from 736 centers were enrolled in the GLORIA-AF Phase II program [22], and between January 2015 and December 2016, 21,300 eligible patients across 931 sites and 38 countries were enrolled in Phase III [19]. This included 1,026 and 1,934 Chinese patients who were enrolled in Phase II and III, respectively, of which 1,018 and 1,911 Chinese patients were eligible and included in the analysis (of eligible patients, 48.3% and 53.3% recruited from university hospitals, 41.4% and 29.8% from specialist centers, 8.7% and 13.9% from general practice/primary care, and 1.6% and 1.0% from community hospitals, respectively; 1.9% of patients in Phase III were recruited from other settings). Almost all prescriptions were issued by cardiologists (99.7% in Phase II; 92.6% in Phase III). The baseline and demographic characteristics are shown in Table 1. The proportion of those with risk factors for stroke and bleeding (such as age, gender, hypertension, diabetes mellitus, previous stroke, previous transient ischemic attack and previous bleeding conditions) [23, 24] were generally similar between Phase II and III (Table 1), except for small differences (standardized difference > 10.0%) in the proportion of patients at low risk of bleeding (HAS-BLED score < 3), patients with ischemic stroke or permanent AF, and patients who received prior AF interventions (cardioversion or cardiac ablation), as well as patients with different AF categorization (Table 1).

**Table 1:** Baseline characteristics of eligible China patients at Phase II and Phase III

	Phase II N = 1,018	Phase III N = 1,911	Standardized difference
Age, mean (SD), yrs	67.5 (11.6)	66.8 (11.1)	–0.0658
Age group, yrs, n (%)			
< 65	393 (38.6)	758 (39.7)	0.0217
65 to < 75	308 (30.3)	647 (33.9)	0.0772
75 to < 80	157 (15.4)	280 (14.7)	−0.0216
80 to < 85	115 (11.3)	163 (8.5)	−0.0927
≥ 85	45 (4.4)	63 (3.3)	−0.0584
Female, n (%)	457 (44.9)	839 (43.9)	−0.0199
BMI*, mean (SD), kg/m^2^	24.73 (3.9)	24.73 (3.4)	−0.0006
History of hypertension, n (%)	660 (64.8)	1259 (65.9)	0.0220
Coronary artery disease, n (%)	363 (35.7)	605 (31.7)	−0.0847
Congestive heart failure, n (%)	306 (30.1)	491 (25.7)	–0.0975
Diabetes mellitus, n (%)	203 (19.9)	440 (23.0)	0.0751
Myocardial infarction, n (%)	119 (11.7)	181 (9.5)	−0.0722
Chronic GI disease, n (%)	63 (6.2)	141 (7.4)	0.0473
Previous stroke, n (%)	115 (11.3)	250 (13.1)	0.0546
Ischemic stroke, n (%)	54 (5.3)	215 (11.3)	0.2171
Hemorrhagic stroke, n (%)	2 (0.2)	13 (0.7)	0.0733
Transient ischemic attack, n (%)	22 (2.2)	47 (2.5)	0.0199
Prior interventions in AF, n (%)			
Cardioversion	139 (13.7)	367 (19.2)	0.1502
Cardiac ablation	79 (7.8)	244 (12.8)	0.1656
Previous bleeding, n (%)	23 (2.3)	51 (2.7)	0.0264
CKD, n (%), CrCl < 60 mL/min	269 (26.4)	442 (23.1)	−0.0764
CHA_2_DS_2_-VASc score, mean (SD)	3 (1.6)	2.9 (1.6)	−0.0443
CHA_2_DS_2_-VASc score class, n (%)			
Low (score = 1 for females)	44 (4.3)	95 (5.0)	0.0308
Moderate (score = 1 for males, 2 for females)	265 (26.0)	496 (26.0)	−0.0017
High (score ≥ 2 for males, ≥ 3 for females)	709 (69.6)	1320 (69.1)	−0.0124
HAS-BLED score, mean (SD)	1.6 (1.1)	1.6 (1.1)	−0.0554
HAS-BLED score class, n (%)			
Low (score < 3)	681 (66.9)	1471 (77.0)	0.2258
High (score ≥ 3)	176 (17.3)	336 (17.6)	0.0077
Missing	161 (15.8)	104 (5.4)	−0.3414
Type of AF, n (%)			
Paroxysmal	613 (60.2)	1206 (63.1)	0.0595
Persistent	392 (38.5)	648 (33.9)	−0.0958
Permanent	13 (1.3)	57 (3.0)	0.1183
Categorization of AF, n (%)			
Symptomatic	231 (22.7)	723 (37.8)	0.3342
Minimally symptomatic	519 (51.0)	819 (42.9)	–0.1634
Asymptomatic	268 (26.3)	369 (19.3)	–0.1678

*BMI missing for patients in Phase III: n = 24 (1.3%)

The standardized difference with absolute value < 0.1 was considered as balanced between groups

AF, atrial fibrillation; BMI, body mass index; CKD, chronic kidney disease; CrCl, creatine clearance; GI, gastrointestinal; SD, standard deviation

### Antithrombotic treatment patterns

**Fig. 2:**
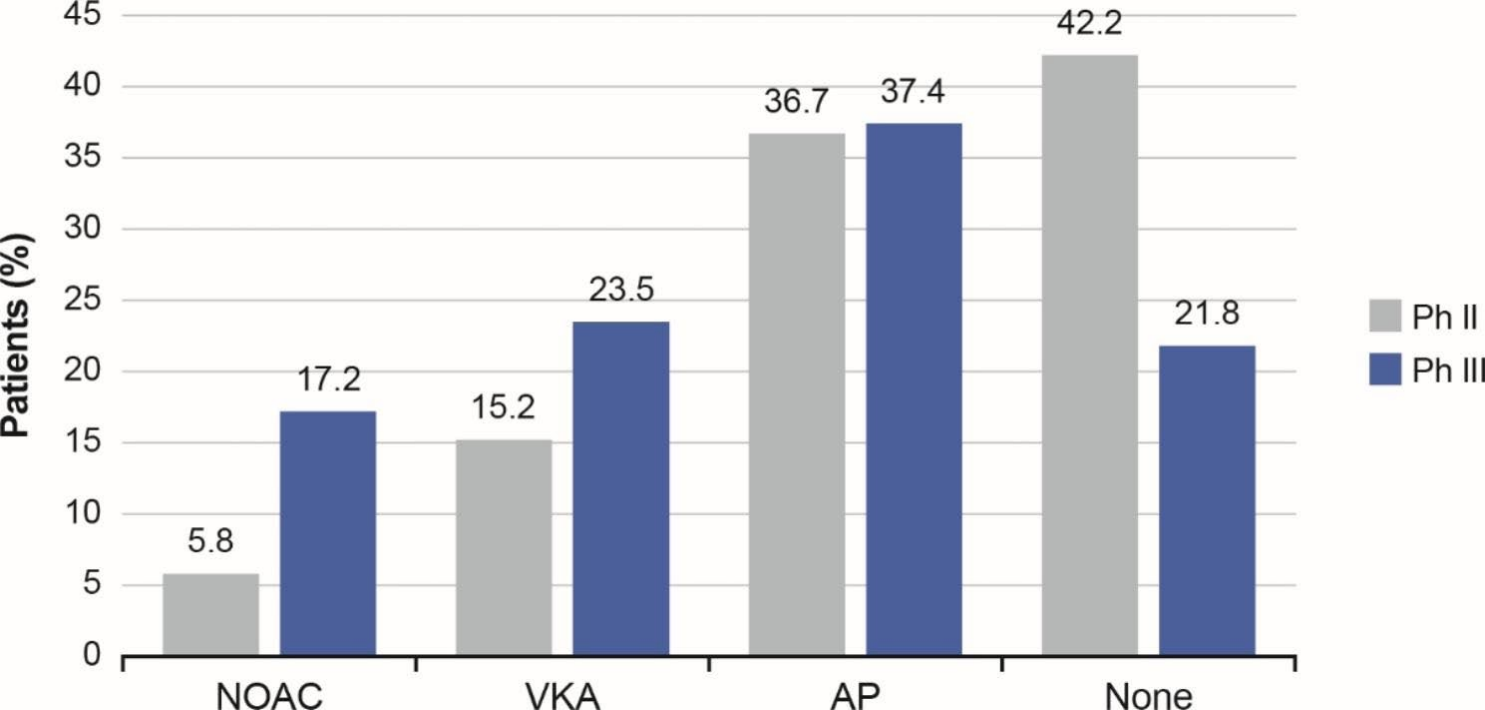
Antithrombotic prescription patterns at baseline in eligible patients from China at Phase III and Phase II. AP, antiplatelet therapy; NOAC, non-VKA oral anticoagulant; VKA, vitamin K antagonist. None indicates no antithrombotic treatment

Antithrombotic treatment patterns at baseline for eligible patients from China in Phase II and Phase III are shown in Fig. 2. OAC use increased from 21.0% in Phase II to 40.8% in Phase III; this increase was greater with NOACs than VKAs. The distributions of prescribed antithrombotic treatments at Phase II were 5.8%, 15.2%, 36.7% and 42.2% for NOACs, VKAs, antiplatelets and no antithrombotic treatment, respectively. For Phase III, the respective treatment distributions were 17.2%, 23.5%, 37.4% and 21.8%. Among NOACs, most patients at Phase II and III were prescribed dabigatran (96.6% and 87.2%) followed by rivaroxaban (3.4% and 12.5%), and only one patient in Phase III received apixaban (0.3%) (Supplemental Table S1).

For patients prescribed dabigatran, the majority were on a lower dose of 110 mg twice daily in Phase II (98.2%) and Phase III (94.8%), respectively, while 1.8% and 2.4% of patients were on a standard dose of 150 mg twice daily (Supplemental Table S1). For rivaroxaban, 50.0% of patients received 15 mg and 50.0% received 20 mg daily in Phase II, while 36.6% received 15 mg and 36.6% received 20 mg in Phase III, respectively. In Phase III, there was also 26.8% of patients who received ‘other dose’ of rivaroxaban and the single patient on apixaban received the lower dose of 2.5 mg twice daily (Supplemental Table S1).

The proportion of patients who used antiplatelets remained stable across Phase II (36.7%) and Phase III (37.4%), and the proportion of patients who received no antithrombotic treatment at Phase III decreased by almost twofold from Phase II (from 42.2 to 21.8%).

### Antithrombotic treatment patterns by risk profiles

**Fig. 3:**
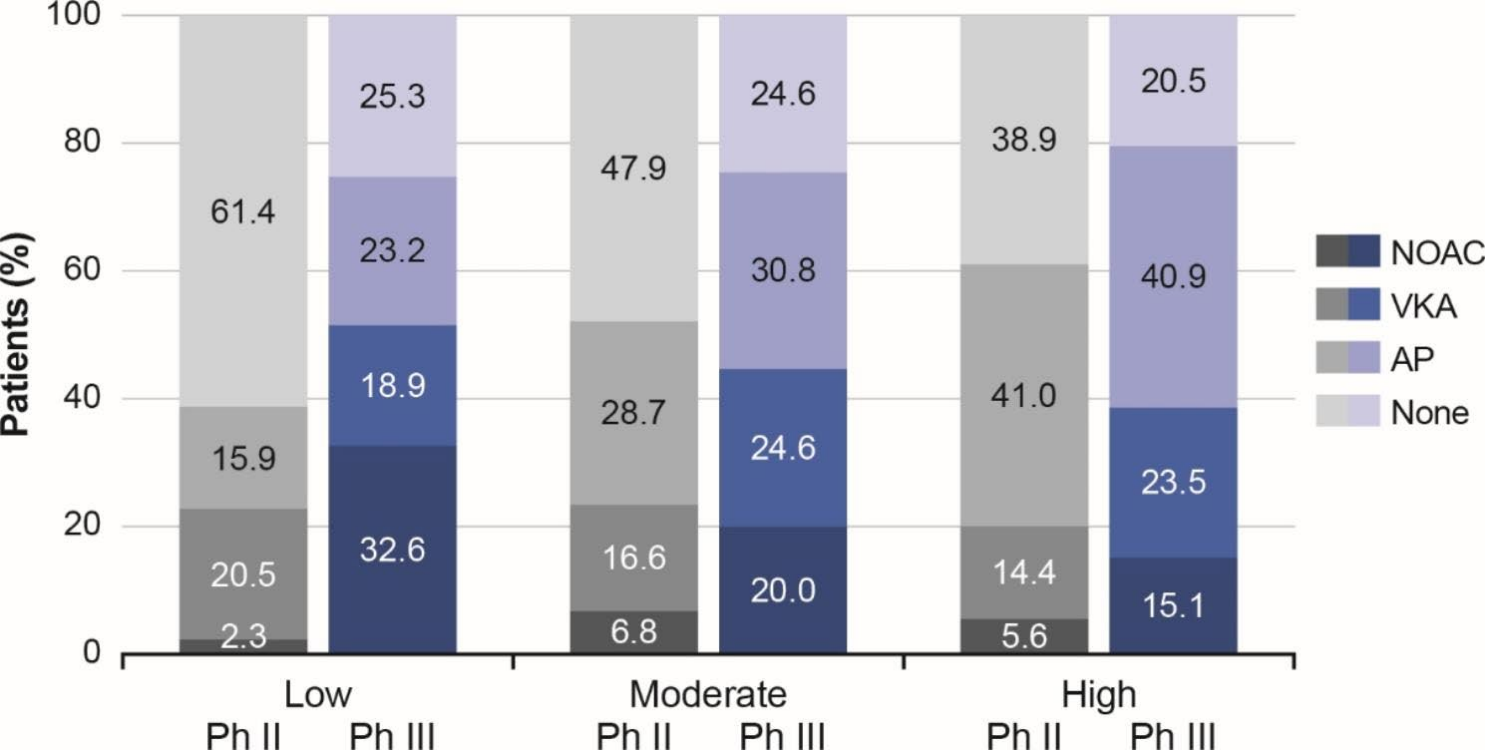
Antithrombotic treatment use by stroke risk in eligible patients from China at Phase II and Phase III. Stroke risk stratification by CHA_2_DS_2_-VASc score: low (score = 1 for females); moderate (score = 1 for males or 2 for females); high (score ≥ 2 for males or ≥ 3 for females). AP, antiplatelet therapy; NOAC, non-VKA oral anticoagulant; VKA, vitamin K antagonist. None indicates no antithrombotic treatment

Treatment distribution according to stroke risk in patients eligible for Phase II and III is shown in Fig. 3. While the trend of OAC prescriptions increased from Phase II to III across patients with low, moderate or high stroke risk, most of these patients remained either on antiplatelets or received no antithrombotic treatment. In patients at high risk of stroke, less than half received OACs (20.0% and 38.6% for Phase II and III, respectively), among whom 5.6% and 15.1% received NOACs. A reduction in the proportion of patients at high risk of stroke who received no antithrombotic treatment was observed at Phase III (20.5%) compared with Phase II (38.9%).

**Fig. 4:**
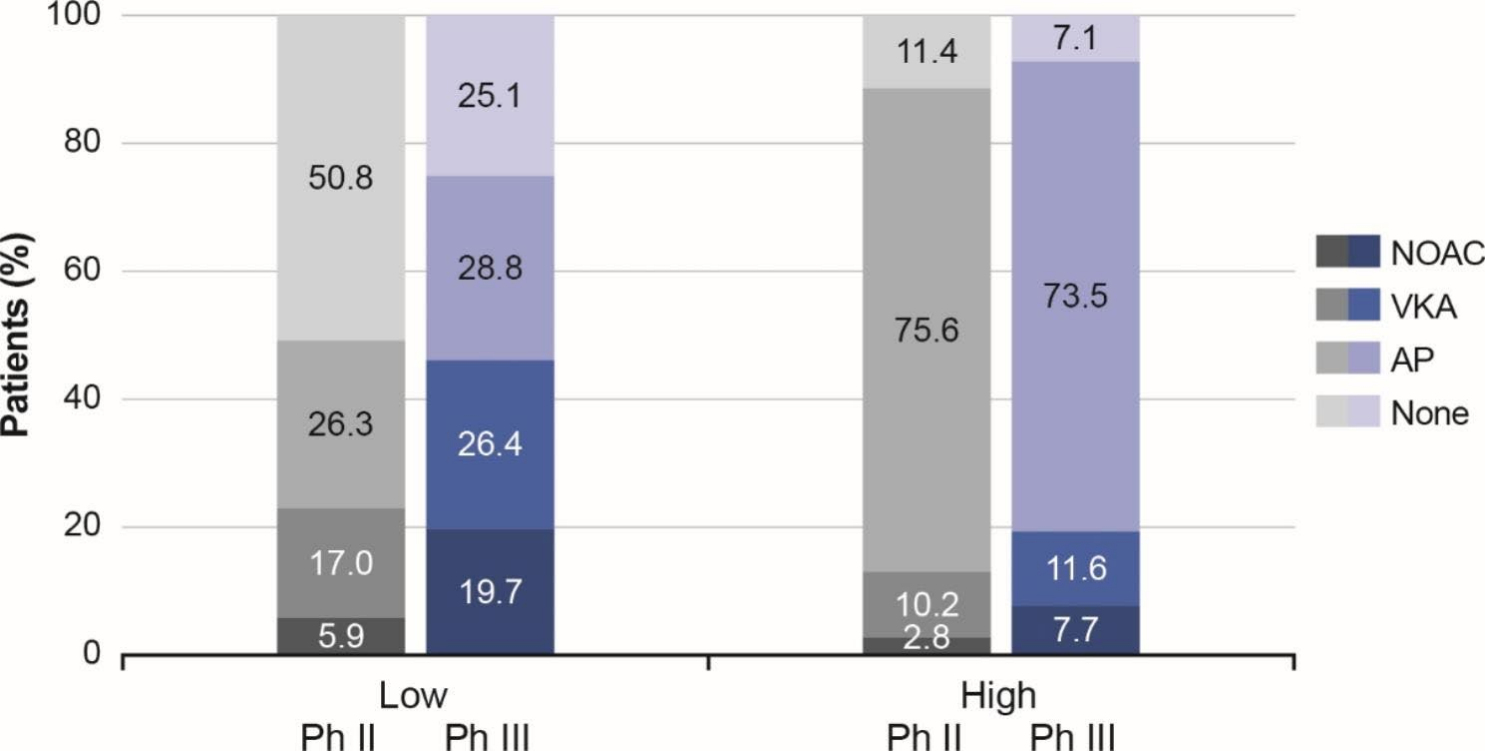
Antithrombotic treatment use by bleeding risk in eligible patients from China at Phase II and Phase III. Bleeding risk stratification by HAS-BLED score: low (score < 3); high (score ≥ 3). AP, antiplatelet therapy; NOAC, non-VKA oral anticoagulant; VKA, vitamin K antagonist. None indicates no antithrombotic treatment

Figure 4 shows the treatment distribution by bleeding risk in eligible patients in Phase II and III. In patients with high bleeding risk, total OAC use was 13.1% at Phase II and 19.3% at Phase III, mainly represented by VKA prescriptions at 10.2% and 11.6%, respectively. The uptake of OACs was observed to increase from Phase II to Phase III in patients with low bleeding risk, at 5.9% and 19.7% for NOACs, and 17.0% and 26.4% for VKAs, respectively.

Antiplatelet therapy remained unchanged as the highest prescribed therapy among patients with high bleeding risk (> 70.0%) regardless of phase, and the trend was also observed to be stable across both phases for patients with low bleeding risk. One-quarter of patients with low bleeding risk did not receive any antithrombotic treatment at Phase III, although this had decreased from 50.8% at Phase II.

## Discussion

This analysis reports the trend of antithrombotic treatment since NOAC therapy was first introduced in China, gradually becoming more widely adopted by physicians. Our observations show a continuously increasing uptake of guideline recommended OAC treatment from Phase II to Phase III among patients with newly diagnosed nonvalvular AF at risk of stroke, including both NOAC and VKA prescriptions, with the latter as the more frequently prescribed OAC therapy overall. This trend was also observed in patients at high risk of stroke, although the proportion of those who received NOACs as first-line therapy for antithrombotic treatment remained relatively low. Furthermore, almost two-thirds of patients with AF remained undertreated at Phase III, either with antiplatelets or no antithrombotic treatment.

OACs are recognized as the standard of care (SoC) for stroke prevention in patients with AF, both internationally [12, 13] and locally [10, 11, 25]. While guideline-adherent therapy of AF has been shown to improve outcomes [26], the management of patients with AF in clinical practice sometimes may differ from evidence-based recommendations. Based on studies of real-world clinical practice between 2011 and 2014, an increase in OAC prescription has been observed in antithrombotic treatment records of patients with nonvalvular AF across China; however, the rates vary by 4.0–68.4% [17, 27]. Our analysis of the GLORIA-AF Phase II and III data reported a continuation of the increase in OAC prescription over time (from 21.0 to 41.0%) among Chinese patients with AF. The shift in treatment distribution had been largely explained by the approximately 50.0% reduction for no antithrombotic treatments (down from 42.0 to 22.0%). However, the landscape of anticoagulation with OACs for stroke prevention in patients with AF in China remains suboptimal compared with the Asia (61.6%) [28] and global (82.2%) [29] GLORIA-AF Phase III populations. The Chinese subgroup in the GLORIA-AF Phase III population was noted to be younger than the overall global GLORIA-AF Phase III population (mean [SD] age 66.8 [11.1] and 70.5 [10.6], respectively) [29] and with a lower body mass index (BMI; mean [SD] 24.73 [3.4] and 28.6 [6.4], respectively) [29]; a lower age and BMI has been associated with a lower probability of OAC prescription, especially in Asia [30]. Other international real-world registries such as GARFIELD-AF and EORP-AF have reported that over two-thirds of patients with AF were receiving OACs [31, 32], and at least one-third to one-half of all patients with AF were receiving OACs in other registries across the East Asia region [33, 34]. Close to 40.0% of Chinese patients with newly diagnosed AF received antiplatelets at Phase III, with minimal change from Phase II, despite a lack of evidence of stroke prevention with antiplatelets [12]. These rates are similar to that of other Chinese AF registries [27, 35] and are of concern, especially among the elderly, whereby antiplatelets are associated with a greater risk of ischemic stroke than VKAs [36, 37].

Of the OAC treatments, NOACs are recommended in preference to VKAs in NOAC-eligible patients [6, 10–13]. This is supported by evidence of greater reduction in risk of stroke or systemic embolic events compared with warfarin use from a meta-analysis of pivotal, randomized controlled trials [38], and real-world data [39]. Similar findings with NOACs have been demonstrated in Asian patients with AF [40], especially a significant reduction with regards to the risk of intracranial bleeding [41, 42]. An increase in NOACs from Phase II to Phase III was observed in Chinese patients with AF, yet the proportion of NOAC usage remains lower compared with the global and Asian GLORIA-AF Phase III cohorts (60.3% and 42.7%, respectively) [29, 43].

Of the NOACs prescribed in both the GLORIA-AF Phase II and Phase III China cohorts, the most common was dabigatran, followed by rivaroxaban; this is in line with the trend of oral anticoagulant use for AF from other local studies of a similar period [44]. The indication of dabigatran for AF was approved in China in 2013, followed by rivaroxaban in 2015, while apixaban is not indicated for AF locally. This contrasts with the earliest approvals of NOACs with dabigatran since 2008 and 2010 in Europe and the USA, respectively. Therefore, we observe a difference in the distribution of NOAC prescriptions versus the GLORIA-AF Phase 3 global cohort, which mainly involved sites from Europe and the USA, where apixaban was the most common NOAC, followed by rivaroxaban, then dabigatran [43].

For patients who received dabigatran, most of the prescriptions in this sub-population analysis is of the lower dose, which is common among Asian patients with AF due to concerns regarding known increased bleeding risk with antithrombotic agents among Asian patients due to smaller body sizes, bleeding tendencies, and lower renal clearance rates [29]. While low dose dabigatran is available for those at risk of bleeding [6, 45–47], studies have also demonstrated standard dose NOACs as more effective and safer in Asians versus non-Asians for eligible patients with AF and has been highlighted as such in local guideline [10]. Therefore, local uptake of NOACs is expected to increase over time with greater understanding of guidelines and improved access.

Guidelines recommend NOAC use in patients with AF at high risk of stroke, as defined by the CHA_2_DS_2_-VASc scale (male: score ≥ 2, female: score ≥ 3) [5, 11–13, 48], which is a clinical risk factor-based scoring system that has been validated in Asian and Chinese populations [49]. Suboptimal guideline adherence for OACs in high-risk patients is evident in China, and NOAC use remains poor in this population [50, 51]. While the proportion of patients at high risk of stroke was lower in the China cohort than the overall global cohort in both GLORIA-AF phases (Phase II: 69.6% and 86.1% [16]; Phase III: 69.1% and 79.0%, respectively) [29], less than half of the Chinese patients with high stroke risk in GLORIA-AF Phase II and III had received OAC treatment. Notably, only 15.1% of Chinese patients with AF at high risk of stroke in Phase III received NOACs, whereas NOAC usage in the general GLORIA-AF Asia cohort (represented largely by South Korea) had reached 67.1% in the fourth year after local NOAC approval [28]. The apprehension towards NOAC uptake in this high-risk population is also reflected in other global registries [31].

Among patients at high risk of bleeding, the proportion who received OAC therapy remained low. Previously, VKAs were the only OAC option, therefore the general reluctance of physicians to prescribe VKAs due to international normalized ratio control challenges could have been overgeneralized to NOACs, though the latter have demonstrated comparatively better safety than VKAs for stroke prevention [52]. High risk of bleeding according to the HAS-BLED score is not a reason to avoid anticoagulation, and guidelines have recommended lower dosage (e.g., dabigatran 110 mg) to accommodate for increased bleeding risk [12]. Considering that the proportion of Chinese patients in GLORIA-AF with a high HAS-BLED score is greater than the global cohort (Phase II: 17.3% and 9.1% [16], Phase III: 17.6% and 9.3% [29]), and that most of those patients did not receive OAC treatment (80.7%), this study highlights an urgent clinical gap for this subpopulation.

Despite increased awareness of AF and recognition of the need for anticoagulation, barriers preventing patients from accessing effective treatment remain a challenge. Possible reasons for the suboptimal use of OACs and NOACs in China include access to SoC treatments, and divergence in clinical practices across the country [17, 27]. VKAs had been the only available OAC in China prior to the approval of dabigatran for stroke prevention in 2013, however the uptake of dabigatran remained slow after local availability, despite more evidence showing an increased risk of bleeding during anticoagulation treatment with VKAs in Asian patients [5, 7, 41, 42]. Physicians’ knowledge of anticoagulation therapy for patients with nonvalvular AF in China (particularly NOACs) may remain inadequate [53]. Findings from a recent cross-sectional study of primary care physicians suggested a positive demand for updated knowledge on AF management, especially with regards to OAC prescriptions [53]. There are also practical challenges in implementing best practice among treating physicians in China. Disparities exist in healthcare standards between different regions and hospitals across China. While there are different medical services from primary healthcare to tertiary or specialist hospitals, the general local population prefers to seek AF-related treatment consultations from the latter. In GLORIA-AF Phase 3, at least 80.0% of participating China-based sites are of university hospitals or specialist centers, and we observe that these tertiary centers report a different antithrombotic use profile than those in general practice/primary care (Supplemental Table S2). Another consideration is that the uptake of anticoagulation may be less likely in rural areas due to cost and accessibility considerations [8, 27], reflecting an ongoing nationwide need for long-term interventions that focus on education, screening, and management of AF [54]. Patient adherence to OAC therapy in China is poor; further understanding of patient preferences on treatment attitudes is important to improve adherence [55]. Broad efforts at improving the prescription of OACs in AF are clearly needed to facilitate uptake and optimize stroke prevention, especially in high stroke risk groups [56].

### Strengths and limitations

The limitations of the GLORIA-AF study design and analysis have been discussed previously [29]; considerations specific to this analysis are listed here. GLORIA-AF is the largest long-term global registry program from 2011 to 2016 that captured the evolution of anticoagulation therapy for stroke prevention in patients with nonvalvular AF worldwide, including China. The broad inclusion of participating sites was representative of those treated within different healthcare settings across different regions in China, and patients were enrolled consecutively to avoid potential selection bias. Approximately half of the sites in China (n = 26) had participated in both Phase II and Phase III (45 sites each), which may have introduced site bias given the difference in site characteristics. Nonetheless, considering that the participating sites in Phases II or III largely comprised university hospitals and specialist centers, similar (tertiary) levels of clinical practice and healthcare infrastructure can be expected. The baseline characteristics of patients in both phases were also mostly comparable with no systematic bias observed; therefore, it can be assumed that the enrollment patterns and patient type in Phase II or Phase III were similar and not influenced by type of recruitment site. Since the aim of GLORIA-AF was to assess real-world outcomes of NOACs with VKA across a broad spectrum of healthcare settings across different countries, the current sampling strategy was not designed to provide a national representation of the anticoagulant prescribing patterns in China.

The design of GLORIA-AF aimed to capture the uptake of antithrombotic therapy over time; therefore, patient enrollment was focused on those eligible for stroke prevention, or CHA_2_DS_2_-VASc score ≥ 1. Stroke prevention is the first step in the Atrial Fibrillation Better Care (ABC) pathway for AF management. GLORIA-AF is essential for monitoring the changes and implementation of updated best practice in clinical care as part of the initiative to enable an integrated approach to AF care [57], and thus better clinical outcomes [58, 59].

Using prospective, consecutively collected local data from Phase III GLORIA-AF, the study has provided evidence that confirm other local regional studies based on retrospective data to monitor the local antithrombotic over similar periods [60, 61]. These studies observed a consistent increase in the uptake of anticoagulants, especially NOACs, demonstrating increased awareness of the best practice of OAC treatment in AF among healthcare practitioners, and could also be largely due to an important change in the local medical system when NOACs were included in the National Drug Reimbursement List in 2017 [62]. Nonetheless, OAC rates in China remain low versus other Asian and international countries [28], and the use of antiplatelet therapy is still prevalent, especially among high-risk populations [56]. Future studies should explore anticoagulation therapy in patients with AF at risk of stroke to further understand the situation with regards to clinical practice in stroke prevention.

## Conclusions

Since the availability of dabigatran in China, the overall trend of OAC, including NOAC prescriptions, in Chinese patients with nonvalvular AF has increased over time, with VKAs as the most commonly prescribed antithrombotic treatment during the timeframe of the study. The use of antiplatelet use in China remained high despite lack of supporting evidence. Overall, most patients, including those at high stroke risk, remain undertreated. Further efforts are required to encourage clinicians and physicians to follow best practice guidelines, which provide evidence-based recommendations for the treatment of AF in newly diagnosed patients.

## Electronic supplementary material

Below is the link to the electronic supplementary material.

Supplementary Material 1

## Data Availability

Please refer to Data Sharing Statement in the Supplementary.

## Abbreviations

ABC: Atrial Fibrillation Better Care
AF: atrial fibrillation
BMI: Body mass index
GLORIA-AF: Global Registry on Long-Term Oral Antithrombotic Treatment in Patients with Atrial Fibrillation
NOAC: Non-vitamin K antagonist oral anticoagulant
OAC: Oral anticoagulant
SoC: Standard of care
VKA: Vitamin K antagonist
