# Supplementary material for: Real world time trends in antithrombotic treatment for newly diagnosed atrial fibrillation in China: reports from the GLORIA-AF Phase III registry: Trends in antithrombotic therapy use in China
Source: Thromb J. 2023 Aug 1;21:83. doi: 10.1186/s12959-023-00527-x (PMC10394786; doi:10.1186/s12959-023-00527-x)
Supplement: Supplementary file 1 — Supplementary Material 1 [file 12959_2023_527_MOESM1_ESM.docx]

**Real world time trends in antithrombotic treatment for newly diagnosed atrial fibrillation in China: Reports from the GLORIA-AF Phase III registry**

Xiaoxia Liu^1*^, Guoze Feng^2^, Sabrina Vogel Marler^3^, Menno V Huisman^4†^, Gregory Y. H. Lip^5†^, Changsheng Ma^1*†^

**Affiliations:**

^1^Department of Cardiology, Beijing An Zhen Hospital, Capital Medical University, Beijing, China. ^2^Boehringer Ingelheim, Shanghai, China. ^3^Boehringer Ingelheim Pharmaceuticals, Inc., Ridgefield, CT, USA. ^4^Department of Thrombosis and Hemostasis, Leiden University Medical Center, Leiden, the Netherlands. ^5^University of Liverpool and Liverpool Heart & Chest Hospital, Liverpool, UK and Aalborg University, Aalborg, Denmark.

*Co-corresponding authors

^†^Menno Huisman and Gregory Lip are co-Chairs of the GLORIA-AF program, and joint senior authors with Changsheng Ma.

**Supplemental Tables**

**Table S1**. NOAC distribution patterns in eligible China patients in Phase II and Phase III of the GLORIA-AF program

|  | Phase II | Phase III |
| --- | --- | --- |
| Overall NOAC, N | 59 | 329 |
| Dabigatran, n (%) |  |  |
| Total* | 57 (96.6) | 287 (87.2) |
| 150 mg BID | 1 (1.8) | 7 (2.4) |
| 110 mg BID | 56 (98.2) | 272 (94.8) |
| 75 mg BID | 0 (0.0) | 1 (0.3) |
| Other dosage | 0 (0.0) | 7 (2.4) |
| Rivaroxaban, n (%) |  |  |
| Total* | 2 (3.4) | 41 (12.5) |
| 20 mg QD | 1 (50.0) | 15 (36.6) |
| 15 mg QD | 1 (50.0) | 15 (36.6) |
| Other dosage | 0 (0.0) | 11 (26.8) |
| Apixaban, n (%) |  |  |
| Total* | - | 1 (0.3) |
| 5 mg BID | - | 0 (0.0) |
| 2.5 mg BID | - | 1 (100.0) |
| Other dosage | - | 0 (0.0) |

*Proportion calculated based on total NOAC patients.
BID, twice daily; GLORIA-AF, Global Registry on Long-Term Oral Antithrombotic Treatment in Patients with Atrial Fibrillation; NOAC, non-vitamin K antagonist oral anticoagulant; QD, once daily.

**Table S2**. Antithrombotic treatment use by medical setting eligible China patients in Phase III of the GLORIA-AF program

|  | Total  N (%) | NOAC  n (%) | VKA  n (%) | AP  n (%) | None  n (%) |
| --- | --- | --- | --- | --- | --- |
| Total | 1911 (100.0) | 329 (17.2) | 450 (23.6) | 715 (37.4) | 417 (21.8) |
| GP/primary care | 266 (100.0) | 22 (8.3) | 47 (17.7) | 154 (57.9) | 43 (16.2) |
| Specialist office | 569 (100.0) | 124 (21.8) | 127 (22.3) | 219 (38.5) | 99 (17.4) |
| Community hospital | 20 (100.0) | 1 (5.0) | 1 (5.0) | 17 (85.0) | 1 (5.0) |
| University hospital | 1019 (100.0) | 181 (17.8) | 261 (25.6) | 305 (29.9) | 272 (26.7) |
| Outpatient healthcare center | 0 (100.0) | 0 (0.0) | 0 (0.0) | 0 (0.0) | 0 (0.0) |
| Anticoagulation clinics | 0 (100.0) | 0 (0.0) | 0 (0.0) | 0 (0.0) | 0 (0.0) |
| Other | 37 (100.0) | 1 (2.7) | 14 (37.8) | 20 (54.1) | 2 (5.4) |

AP, anti-platelet; GP, general practitioner; VKA, vitamin K antagonist.

**Data Sharing Statement**

To ensure independent interpretation of clinical study results, Boehringer Ingelheim grants all external authors access to all relevant material, including participant-level clinical study data, and relevant material as needed by them to fulfill their role and obligations as authors under the ICMJE criteria.

Furthermore, clinical study documents (e.g., study report, study protocol, statistical analysis plan) and participant clinical study data are available to be shared after publication of the primary manuscript in a peer-reviewed journal and if regulatory activities are complete and other criteria met per the BI Policy on Transparency and Publication of Clinical Study Data: <https://trials.boehringer-ingelheim.com/>

Prior to providing access, documents will be examined, and, if necessary, redacted and the data will be de-identified, to protect the personal data of study participants and personnel, and to respect the boundaries of the informed consent of the study participants.

Clinical Study Reports and Related Clinical Documents can also be requested via the link <https://trials.boehringer-ingelheim.com/>

All requests will be governed by a Document Sharing Agreement.

Bona fide, qualified scientific and medical researchers may request access to de-identified, analyzable participant clinical study data with corresponding documentation describing the structure and content of the datasets. Upon approval, and governed by a Data Sharing Agreement, data are shared in a secured data-access system for a limited period of 1 year, which may be extended upon request.

Researchers should use the <https://trials.boehringer-ingelheim.com/> link to request access to study data.
